# Pooled PCR testing strategy and prevalence estimation of submicroscopic infections using Bayesian latent class models in pregnant women receiving intermittent preventive treatment at Machinga District Hospital, Malawi, 2010

**DOI:** 10.1186/1475-2875-13-509

**Published:** 2014-12-18

**Authors:** Zhiyong Zhou, Rebecca Mans Mitchell, Julie Gutman, Ryan E Wiegand, Dyson A Mwandama, Don P Mathanga, Jacek Skarbinski, Ya Ping Shi

**Affiliations:** Malaria Branch and Division of Parasitic Diseases and Malaria, Center for Global Health, Centers for Disease Control and Prevention, Atlanta, GA USA; Malaria Alert Centre, University of Malawi College of Medicine, Blantyre, Malawi

**Keywords:** Submicroscopic malaria infection, Nested PCR, Placental histology, IPTp, Pooled sample estimates, Latent class models (LCMs)

## Abstract

**Background:**

Low malaria parasite densities in pregnancy are a diagnostic challenge. PCR provides high sensitivity and specificity in detecting low density of parasites, but cost and technical requirements limit its application in resources-limited settings. Pooling samples for PCR detection was explored to estimate prevalence of submicroscopic malaria infection in pregnant women at delivery. Previous work uses gold-standard based methods to calculate sensitivity and specificity of tests, creating a challenge when newer methodologies are substantially more sensitive than the gold standard. Thus prevalence was estimated using Bayesian latent class models (LCMs) in this study.

**Methods:**

Nested PCR (nPCR) for the 18S rRNA gene subunit of *Plasmodium falciparum* was conducted to detect malaria infection in microscopy-negative Malawian women on IPTp. Two-step sample pooling used dried blood spot samples (DBSs) collected from placenta or periphery at delivery. Results from nPCR and histology as well as previously published data were used to construct LCMs to estimate assay sensitivity and specificity. Theoretical confidence intervals for prevalence of infection were calculated for two-step and one-step pooling strategies.

**Results:**

Of 617 microscopy-negative Malawian women, 39 (6.3%) were identified as actively infected by histology while 52 (8.4%) were positive by nPCR. One hundred forty (22.7%) individuals had past infection assessed by histology. With histology as a reference, 72% of women in the active infection group, 7.1% in the past infection group and 3.2% in histology-negative group were nPCR positive. Using latent class models without a gold standard, histology had a median sensitivity of 49.7% and specificity of 97.6% for active infection while PCR had a median sensitivity of 96.0% and specificity of 99.1%. The true prevalence of active infection was estimated at 8.0% (CI: 5.8-10.5%) from PCR. PCR also had similar sensitivity for detecting either peripheral or placental malaria for submicroscopic infections. One-step pooling would give similar confidence intervals for pool sizes less than 20 while reducing the number of tests performed.

**Conclusions:**

Pooled nPCR testing was a sensitive and resource-efficient strategy and LCMs provided precise prevalence estimates of submicroscopic infections. Compared to two-step pooling, one-step pooling could provide similar prevalence estimates at population levels with many fewer tests required.

**Electronic supplementary material:**

The online version of this article (doi:10.1186/1475-2875-13-509) contains supplementary material, which is available to authorized users.

## Background

Each year, approximately 50 million women living in malaria-endemic countries throughout the world become pregnant, of whom over half live in tropical areas of Africa with intense transmission of *Plasmodium falciparum* [1]. Pregnant women are at a higher risk of malaria infection than non-pregnant women. Public health consequences of malaria infection during pregnancy include severe maternal anaemia, prematurity and low birth weight of babies which subsequently contribute to increased maternal and neonatal deaths [1–6]. The use of insecticide-treated bed nets, intermittent preventive treatment in pregnancy (IPTp) and case management are recommended by WHO to prevent the adverse consequences of malaria infection among pregnant women living in areas of stable transmission of *P. falciparum* [7, 8]. IPTp reduces the prevalence of malaria and associated severe consequences [9–11]. With a decrease in the prevalence of microscopy-positive *P. falciparum* infection due to preventive interventions, submicroscopic *P. falciparum* infections become relatively more common among pregnant women [12]. Although conflicting results have been obtained, some studies suggest that submicroscopic infections are associated with placental malaria, maternal anaemia [13, 14] and infant low birth weight [15]. Submicroscopic infections are also contributors to transmission in areas with low transmission intensity [16].

A recent review of prevalence estimation using a series of testing modalities clearly demonstrates limited ability to calculate true prevalence of malaria infections in pregnant women using gold-standard based approaches [17]. Diagnosis of malaria in pregnancy is complicated by absent or low parasite densities in peripheral blood due to sequestration of *P. falciparum* in the placenta as well as submicroscopic infections in placenta after IPTp and other interventions [13]. Histological examination of the placenta is considered the gold standard for diagnosis of placental malaria at delivery, but it is frequently not available in the field [18, 19]. Polymerase chain reaction (PCR)-based molecular diagnostic methods for malaria infection are more sensitive and specific compared to microscopy and rapid diagnostic tests [17, 20–22]. The estimated prevalence of placental malaria varies across study populations depending on the population characteristics and the diagnostic methods used [23]. Because PCR is always evaluated relative to another test, assessments of prevalence based on PCR results are tempered by apparent low specificity as an artifact of gold-standard assumptions. However, studies in which multiple testing modalities with limited correlation are used [13] allow estimation of test characteristics using latent class models (LCMs). These models assume that true infection status is unknown (latent) and that neither test is a gold standard, which can perfectly classify positive and negative individuals [24, 25]. Test sensitivity and specificity characteristics estimated using a latent class approach can then be applied to other datasets for prevalence estimation in other populations.

Although the PCR-based diagnostic method for malaria offers increased sensitivity and specificity relative to other diagnostic methods, it requires longer time for sample processing, specialized staff and equipment, and higher reagent cost per sample compared to microscopy and rapid diagnostic tests. Cost-efficient strategies of pooled testing for estimation of population prevalence are necessary, particularly in the areas where prevalence of malaria infection is low and submicroscopic infections are common. Pooled testing strategies are routinely used in estimation of rare infections for public health programmes and estimation of infection prevalence for animal agriculture and for vector surveillance strategies [26–30]. These strategies can be employed for identifying specific infected individuals [31] or to track the prevalence of infection at a population level and detect introduction of new pathogens into populations [29]. Pooled sampling strategies can either involve one-step pools, where prevalence and confidence intervals are calculated based on pool results [26], or multiple-step pools, where individual samples from a positive pool are retested to identify the number of positive individuals per pool [31, 32]. Two-step pooling with re-testing of all individuals from a positive pool is the simplest strategy for multiple-step pooling. Multiple-step pooling methods require more tests than one-step pools while offering only limited increases in estimate precision relative to individual testing [32]. In addition to decreasing costs, pooled testing can improve precision of estimates when imperfect tests are used [30]. Currently, most malaria diagnostic strategies have not used one-step pooled sampling techniques.

The pooling of samples could be a practical and effective means to allow for a larger sample size while reducing the reagent and processing costs, particularly for surveys in areas where malaria prevalence is low and submicroscopic infections are common. Before implementing a systematic strategy of pooling samples, both test characteristics (sensitivity and specificity) and expected prevalence must be well understood. The multiple-step pooling of sera and dried blood spot (DBS) samples has been reported to improve the cost-effectiveness of malaria prevalence estimation [20, 33, 34]. However, the impact of pooling samples on estimate precision is not commonly assessed.

The present study had two main objectives: first, to estimate the sensitivity and specificity of pooled PCR and the prevalence of submicroscopic malaria infection in pregnancy, and second, to evaluate the use of pooled PCR as a potential surveillance option given estimated test characteristics by LCMs. For this work, malaria prevalence estimates at delivery in microscopy-negative Malawian women on IPTp were compared for pooled and individual nested PCR samples against placental histology when assuming neither PCR nor histology is a true gold standard using LCMs. Following calculation of prevalence from a two-step pooled sampling strategy, a one-step pooled-sampling algorithm was applied to calculate the prevalence of malaria infections based on the results obtained from pooled DBS samples rather than individual tests.

## Methods

### Study samples

As part of a study to monitor the effectiveness of IPTp-SP in Malawi, the samples for current study were collected from March 1 to August 30, 2010 at the delivery ward of Machinga District Hospital in southern Malawi. All study protocols were approved by the ethical review boards of the University of Malawi, College of Medicine (Blantyre, Malawi) and the Centers for Disease Control and Prevention (Atlanta, USA). Written informed consent was obtained from all participating women. The details of the IPTp-SP effectiveness study have been described elsewhere [35]. Briefly, peripheral and placental blood at delivery were collected. Thick blood films were prepared from both peripheral and placental blood. Blood smears were stained with Field’s Stain A and B (azure dye and eosin) or Giemsa only. DBS samples were prepared by spotting 50 μl of whole blood onto each of the five circles on a Whatman 903 filter paper (Whatman Inc, Piscataway, NJ) and drying overnight at ambient temperature. Each DBS card was packaged in a Ziploc bag containing desiccant packs and a humidity indicator card and sealed tightly. The packaged DBS cards were stored at room temperature.

### Placenta histology examination

Placental histology examination was conducted in the College of Medicine, University of Malawi. Immediately after delivery, placental biopsy specimens were prepared from placental tissue and placed into 10% neutral buffered formalin solution. After fixation they were embedded in paraffin wax by standard techniques. The sections were stained with haematoxylin-eosin stain. All slides were read by skilled pathologists according to previously established criteria [36, 37]. Acute infection was defined as the presence of parasites only; chronic infection was defined as the presence of both parasites and malaria pigment; past infection was defined as the presence of malaria pigment only in fibrin or monocytes; no infection was defined as the absence of any malaria parasites or pigment in placental tissue. For descriptive results and latent class models, the histologic results were transformed into active infection (acute and chronic infection) or negative (past and no infection) [17].

### DNA extraction with two-step pooling

The DBS cards were shipped to the Malaria Branch laboratory at the Centers for Disease Control and Prevention, Atlanta at ambient temperature. As not all women had both peripheral and placental samples available, a single peripheral or placental sample was processed from each woman. In total, 617 available samples from microscopy-negative women were pooled and examined. Among the 617 samples, 267 (43%) were from peripheral blood and 350 (57%) were from placental blood.

**Figure 1:**
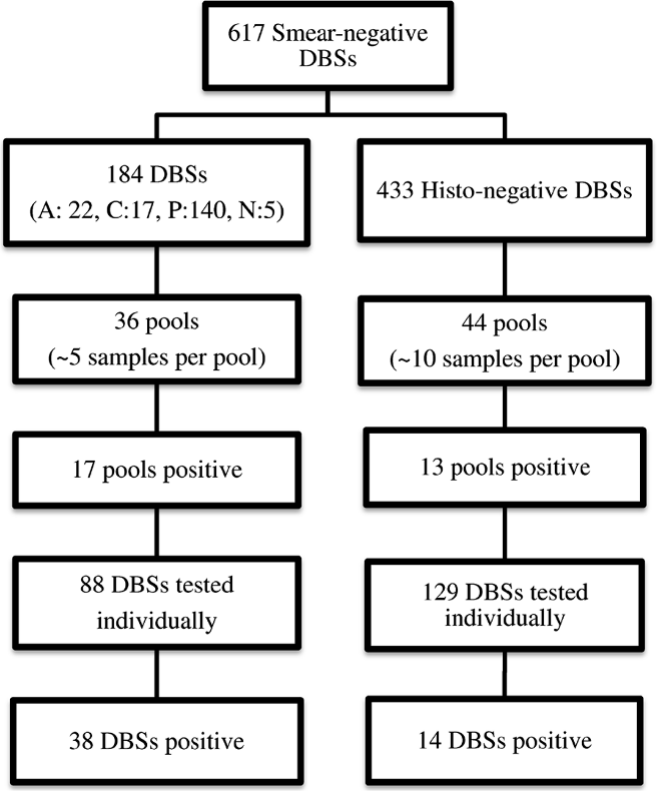
**Pooling strategy for DNA extraction and PCR screening of smear-negative samples from Malawi.** A total of 617 smear-negative samples were used for pooling based on the histology data from the Malawian IPTp effectiveness study. The histology-positive group including acute (A), chronic (C), and past infections (P) by histology was divided into 36 pools with 5 DBS samples per pool. The histology-negative (no infection by histology) group was divided into 44 pools with 10 samples in each pool. Several pools were short one or extra one sample to accommodate all available samples into pools. Five histology-negative samples (N) were processed in the smaller pools due to misclassification. After first round PCR screening, individual DBSs from positive pools were extracted, and second round PCR assays were performed.

Two-step pooling was used to estimate prevalence of malaria in the smear-negative samples. Two-step pooled testing relies on the assumption that the result of the pooled test is perfectly correlated to results of the second set of individual tests. In this case, all individuals from negative pools are considered test negative. This was based on a pilot showing an agreement between the pooled results and individual samples. Pools of 10 or 5 samples were constructed by histologic status (histology-negative *versus* acute/chronic/past infection) (Figure 1). Among the individuals without histologic evidence of infection, pools of 10 were assigned based on low expected prevalence. Among individuals with histologic evidence of infection, pools of five were assigned based on higher expected prevalence. Five samples were misclassified as active infections during pooling without histologic evidence of infection.

One paper disk (5 mm) was punched out from each DBS card and pooled together for DNA extraction. Pooled DBSs were extracted with QIAGEN Mini DNA kit as per manufacturer protocol (QIAGEN, Valencia, CA) and tested using nPCR. For any pool with positive PCR results, individual DBSs were extracted and PCR assays were performed.

### nPCR detection of the 18S small subunit ribosome RNA (rRNA) gene of *P. falciparum*

Purified DNA templates were used for amplification of the 18S rRNA gene subunit of *P. falciparum* using a modified method and *P. falciparum* specific primer set as previously described [38, 39]. Briefly, the 50 μl PCR reaction contained 1× PCR Master Mix (Promega, Madison, WI) and 400 nM of each PCR primer. For each primary PCR reaction, 5 μl of DNA were used. The reactions were performed under the conditions of initial denaturation at 95°C for 5 min, 25 cycles at 94°C for 1 min, annealing at 58°C for 2 min, and final extension at 72°C for 5 min. Secondary PCR was performed as primary PCR except for use of 35 cycles and 2 μl primary PCR product per reaction. The amplified DNA products were stained with GelRed (Biotium, Hayward, CA) in 1.5% agarose gel electrophoresis. The expected DNA band size was 205 base pairs.

### Latent class models (LCMs) for sensitivity and specificity estimation

Data management was performed in SAS v9.3 (SAS Corporation, Cary, NC) and Microsoft Excel 2010 (Microsoft, Redmond, WA). To estimate sensitivity and specificity of the PCR-based technique for detection of malaria infection at delivery, LCMs were implemented in OpenBUGS, v3.2.3 (OpenBUGS Foundation, Helsinki, Finland), R v3.0.2 (R Foundation, Vienna, Austria) and R Studio v0.98.490 (RStudio, Boston, MA USA). The BetaBuster programme (UC Davis, Davis CA) was used to obtain values for specified priors. Estimates were calculated using two-population models which combined data from this study and the data from previous published work from Mozambique [13] in order to increase external validity as well as precision of estimates and identifiability of models. In Mozambique, multiple diagnostic tests provided different prevalence estimates for placental malaria [13]. For data from the current Malawi study in which two-step pooling resulted in only samples from positive pools receiving individual PCR tests, samples from negative pools were assumed to have negative individual tests (Figure 1). To differentiate test characteristics between placental and peripheral samples, two sub-analyses were run for placental and peripheral samples, respectively.

For latent class model estimation, a prior assumption of test characteristics and prevalence is supplied to the programme as well as the available data, and posterior estimates are calculated based on this combination. Because the sensitivity and specificity of PCR have not been calculated by this method previously, a uniform distribution between zero and one can be used as the prior assumption. In this case, uniform prior probability distributions (priors) were used for PCR characteristics and prevalence. Priors used for histology were selected to reflect previous assumptions of high specificity and sensitivity for the method but with broad distributions to allow evaluation of this assumption. To gauge the effect of prior specification on estimates, models were also run with pessimistic priors for PCR, based on previous assessments and realistic prevalence priors (median 20%, 97.5th percentile estimate of 45%) from published studies [17] (Additional file 1). Model output is presented as median and 95% credible intervals. Models were run in OpenBUGS for 130,000 iterations of which the first 30,000 iterations were discarded as burn in. Runs were thinned by a factor of 10 to eliminate autocorrelation in results. Five chains were run for each model to assess convergence, with initial values randomly selected within 0.2 of expected outcome. Mixing within chains and convergence were evaluated graphically and through Gelman-Rubin scale reduction factors [40].

### Prevalence estimation

Median sensitivity (Se) and specificity (Sp) estimates from the LCMs were used to evaluate prevalence based on individual tests and pooled sampling strategies. From original apparent prevalence, true prevalence based on individual sampling was calculated using Rogan-Gladen correction for test characteristics and Clopper-Pearson exact confidence intervals [41]. Expected confidence intervals for overall prevalence based on a one-step pooling strategy was calculated as in Method 5 of Cowling *et al*. [26]. For one-step pooled estimates, confidence limits for individual level prevalence (π) was calculated from expected pooled prevalence given observed individual tests results (P = x/m), the number of positive pools (x) over total number of pools (m). Size of pool (k) influenced confidence intervals for individual-level prevalence. Bounds for number of positive pools were calculated using the inverse of the beta cumulative distribution function (beta.inv) formula in Excel from the  or 1-  percentiles of a beta distribution with shape parameters as shown (Equations 2 and 3). To calculate confidence limits, the lower and upper bound for number of positive pools (P_L_ and P_U_, respectively) were substituted into equation 1 which was then solved for P. If the test was assumed to be perfect (*Se* = *Sp* = *1*), the equation was simplified accordingly.
123

### Estimated number of PCR tests performed

Expected number of positive pools was calculated for pool sizes from 2 to 30 by solving equation 1 for x using test characteristics estimated by the LCMs. Expected number of positive pools was rounded up to the next integer value to calculate total tests performed in two-step strategies (m pools + m*k individual tests). The ratio of tests performed relative to individual testing strategies was plotted for both one-step and two-step testing strategies.

## Results

### Detection of malaria infection

A total of 7.7% of the 703 women enrolled in the IPTp effectiveness study had a positive placental or peripheral smear. Of the 617 microscopy-negative samples, 39 had active infections (22 acute and 17 chronic infections), 140 had past infections, and 438 had no evidence of infection by histology (Table 1). Overall, nPCR detected 52 (8.4%) malaria infections from these microscopy-negative individuals: 16 of 22 (73%) with acute infections by histology, 12 of 17 (71%) with chronic infections, 10 of 140 (7.1%) with past infections, and 14 of 438 (3.2%) with no infection. Separated by blood source, nPCR detected 13 of 17 active infections (76%) from peripheral samples and 15 of 22 active infections (68%) from placental samples (Table 1).

**Table 1:** **PCR results compared to histology findings**

Histological status	Overall	Peripheral	Placental
	PCR (+)/total (%)	Positive/total (%)	Positive/total (%)
**Active**	**28/39 (71.8%)**	**13/17 (76.5%)**	**15/22 (68.2%)**
*Acute*	*16/22 (72.7%)*	*7/10 (70%)*	*9/12 (75%)*
*Chronic*	*12/17 (70.6%)*	*6/7 (85.7%)*	*6/10 (60%)*
**Negative**	**24/578 (4.2%)**	**17/250 (6.8%)**	**7/328 (2.1%)**
*Past infection*	*10/140 (7.1%)*	*7/74 (9.5%)*	*3/66 (4.5%)*
*No infection*	*14/438 (3.2%)*	*10/176 (5.7%)*	*4/262 (1.5%)*

Proportion of PCR positives in each histologic classification is presented for each sample type analysed: overall and by blood sources (peripheral or placental). Histologic results are separated into active infection (acute and chronic) and negative (past infection or no infection).

### Sensitivity and specificity estimation

When histology was assumed to be a perfect gold standard for diagnosis of malaria in pregnancy, sensitivity of nPCR for active infection (acute and chronic) in microscopy-negative individuals was 71.8% (28/39) and specificity was 95.8% (554/578) (Table 2). If LCMs were used with uniform priors, overall, median sensitivity (96.0%, credible interval: 86.2-99.8) and specificity (99.1%, credible interval: 96.9-100) of PCR for detection of malaria infection at delivery was higher than that calculated using the histologic gold standard. Median specificity of PCR was 95% or greater for all simulations (Table 3). However, estimated sensitivity of histology for placental malaria is significantly lower than expected given its treatment as a gold standard, with a median of 50%. Specificity for histology remained high across all models, with median estimates >95.9% (Table 3).

**Table 2:** **Test characteristics as calculated with histology as a gold standard**

	Histology	Histology	Total
	(+)	(-)	
**PCR (+)**	28	24	52
**PCR (-)**	11	554	565
**Total**	39	578	617
**Sensitivity**	71.8%		
**Specificity**		95.8%

**Table 3:** **Posterior probability table for latent class models (LCMs) using uniform priors for PCR characteristics and prevalence**

Value	Uniform ^a^	Uniform	Uniform
		Placental ^b^	Peripheral ^c^
Sensitivity Histology (Median, CI)	0.497 (0.414, 0.588)	0.498 (0.410, 0.589)	0.504 (0.397, 0.646)
Sensitivity PCR (Median, CI)	0.960 (0.862, 0.998)	0.961 (0.859, 0.998)	0.953 (0.841, 0.998)
Specificity Histology (Median, CI)	0.976 (0.963, 0.987)	0.972 (0.955, 0.985)	0.971 (0.951, 0.986)
Specificity PCR (Median, CI)	0.991 (0.969, 0.9996)	0.994 (0.974, 0.9997)	0.964 (0.906, 0.998)
Prevalence Mozambique (Median, CI)	0.368 (0.305, 0.436)	0.370 (0.301, 0.437)	0.334 (0.252, 0.412)
Prevalence Malawi (Median, CI)	0.0813 (0.056, 0.110)	0.063 (0.039, 0.094)	0.094 (0.049, 0.150)

^a^uses all data from the current Malawi study and placental data from published work in Mozambique [13], ^b^uses only placental data from both countries, and ^c^uses only peripheral data from both countries. Values presented are median values of posteriors and 95% credible intervals. The prevalence estimate based on the test characteristics are presented in last two rows.

**Figure 2:**
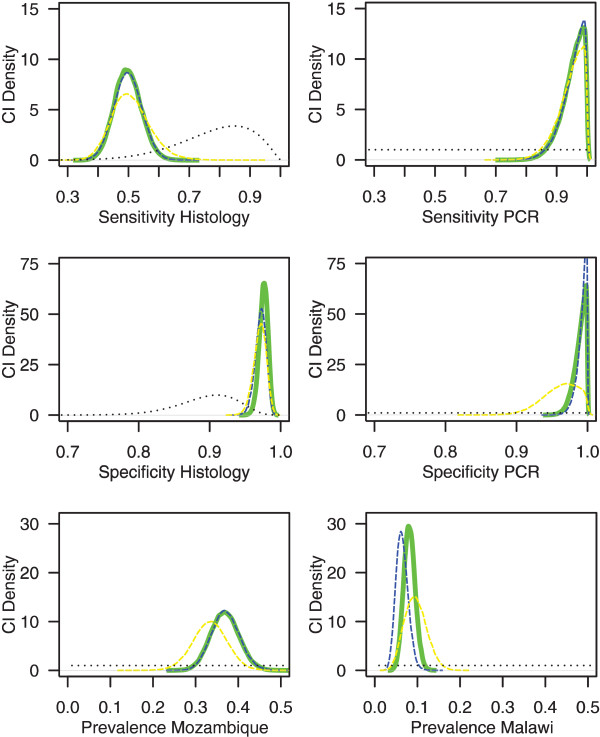
**Density plots for parameter estimates using uniform priors for PCR characteristics and prevalence.** Horizontal axes represent parameter evaluated, and vertical axes represent density of credible intervals. Dashed black lines represent priors. Overall results using all type samples from Malawi and placental samples from Mozambique [13] (thick green lines) compared to result for placental samples only in both countries (thin dashed blue lines) or peripheral samples only in both countries (thin dashed yellow lines).

If the samples drawn from peripheral and placental blood were analysed separately, estimated medians and credible intervals of test characteristics did not change substantially (Table 3). PCR specificity had a lower median value in peripheral blood (96%) compared to estimates generated using all samples or placental blood (>99%), but 95% credible intervals for estimates from peripheral blood (90.6%, 99.8%), placental blood (97.4%, 99.9%) or all samples regardless of source (96.9%, >99.9%) overlapped substantially (Figure 2 and Table 3) not detecting a significant difference between the two populations.

For all LCMs, chains were well mixed, and there was limited autocorrelation among samples. Separate chains generated using randomly-selected initial conditions converged to the same endpoint distributions. Gelman-Rubin scale reduction factors were all less than 1.005 at endpoints indicating convergence of chains. Results from models run using pessimistic PCR priors are available in Additional files 2 and 3.

### Prevalence estimate between pooled and individual samples in Malawi

**Figure 3:**
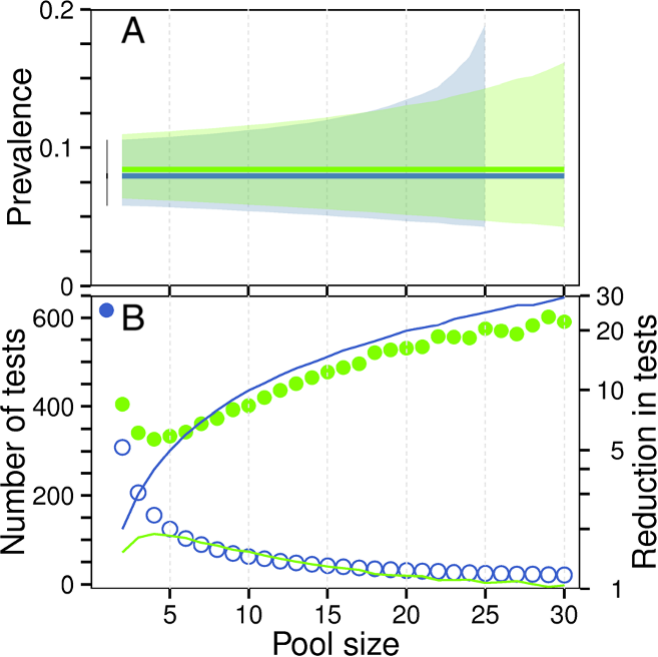
**Predictive relationship between pool size, number of tests and confidence intervals. Panel A**: Expected point estimate and confidence interval for pools of sizes 2 through 30. Blue line and band represent point estimate and confidence interval for an imperfect test based on the values calculated in the LCM (True Prevalence: 8.0%, Sensitivity: 96.0%, Specificity: 99.1%). Confidence interval is not shown for pool sizes where confidence interval includes 100% prevalence (pool sizes >25). Green line and shaded area represent point estimate and confidence interval for a perfect test as would be assumed setting PCR as a gold standard (true prevalence 8.4%, sensitivity: 100%, specificity: 100%). Point estimate and confidence intervals for samples processed individually using imperfect test characteristics represented in black. **Panel B**: Expected number of tests required for one-step and two-step pooling strategies by pool size. Closed blue circle represents individual testing. Open blue circles represent expected number of tests from randomly-mixed one-step pools with an imperfect test. Closed green circles represent expected number of tests from two-step pools with an imperfect test. Reduction in number of tests relative to individual testing (individual tests/pooled tests) is represented for two-step pooling (green solid line) and one-step pooling (blue solid line).

Based on the two-step pooling used in this study, apparent prevalence of malaria infection at delivery among smear-negative samples by nPCR was 8.4% (52/617, CI: 6.4-10.9%). The LCM using all samples produced median prevalence of 8.1 and 95% credible interval from 5.6 to 11% for Malawi. If models were restricted to only placental samples or peripheral samples, prevalence estimates in Malawi overlapped (median: 6.3, CI: 3.7-9.4% *versus* median 9.4, CI: 4.9-15%, respectively) (Table 3). To compare individual and pooled confidence intervals, prevalence was generated using the Rogan-Gladen estimator and Clopper-Pearson confidence intervals. Point prevalence was 8.0%, and Clopper-Pearson confidence intervals increased marginally (CI: 5.8-10.5%) using this method. If randomized one-step pools had been constructed with expected true prevalence of 8.0%, confidence intervals using imperfect tests with sensitivity and specificity calculated here would be 5.6%-10.7% for pools of 5, 5.4%-11.2% for pools of 10, or 4.6%-13.4% for pools of 20 (Figure 3A, blue line and shaded area). With a prevalence of 8.0%, expected number of tests performed to evaluate all 617 samples via randomized pools would be 124 (one-step) or 329 (two-step) tests for pools of 5, 62 (one-step) or 402 (two-step) tests for pools of 10, and 31 (one-step) or 511 (two-step) tests for pools of 20 (Figure 3B). Given the imperfect test characteristics, one-step pools of greater than 25 samples would result in too many positive samples to provide an adequate upper bound on confidence intervals as there is a high probability that all pools would test positive. If estimates were calculated based on assumptions of a perfect gold-standard test, the confidence intervals would be narrower at all pool sizes (Figure 3A, green line and shaded area).

### Estimated number of PCR tests performed

The overall number of tests required for one-step pooling decreases from 309 to 24 tests as pool size increases from 2 to 25 (Figure 3B, open blue circle). The increase in confidence interval width is 1.10×, 1.26× and 1.89× for pools of 5, 10 and 20, respectively (Figure 3A). However, two-step pools, while providing a consistent precise confidence interval, do not result in the same dramatic decrease in number of tests performed, with more than 300 tests performed at any given pool size (Figures 3A, green shaded area and 3B, closed green circle). For this dataset, the maximum reduction in tests performed was 1.84-fold relative to individual testing using a two-step strategy with pools of size 5 (Figure 3B). Reduction in tests performed increased linearly with pool size with one-step testing strategies, with the trade-off of simultaneous widening of confidence intervals (Figure 3A).

## Discussion

In the present study, blood smear-negative DBS samples collected at delivery from Malawian women on IPTp were screened using a two-step pooling strategy of DNA extraction and nested PCR technique to determine submicroscopic infections. The sensitivity and specificity of PCR and true prevalence of submicroscopic malaria infection were estimated using LCMs. PCR had higher sensitivity and specificity than histology in detecting active submicroscopic infection. The sensitivity of PCR was similar in detecting submicroscopic peripheral and placental malaria infections. Pooled sampling strategies using PCR are not only resource–efficient but also provide precise estimates of the population prevalence of active malaria infection.

Prevalence of submicroscopic infection in this study was estimated at 8.0% of smear-negative individuals, confirming that microscopic diagnosis underestimated placental infection in pregnant women due to its limited sensitivity [13, 17, 19]. Submicroscopic infections are common in Malawi and have been associated with decreased maternal hemoglobin [14]. Submicroscopic infections are also potentially important in malaria transmission [16, 21, 42–44]. These infections may happen at any point during pregnancy and would not be detected until delivery without the use of PCR. Thus, surveillance in pregnant women attending first antenatal care by using pooled PCR testing would not only assess population prevalence of malaria infections in pregnancy, but might also serve as a sentinel, before IPTp and other interventions are delivered to pregnant women, for estimating malaria transmission. Although histology can detect past placental infections (an indicator of malaria infection during pregnancy), it is not feasible as a routine diagnostic method in most resource-limited settings. It is technically difficult to differentiate true parasites and artifacts, and true malaria pigment (haemozoin) and artificial formalin pigment in the placental tissue sections [13], which may explain the imperfect sensitivity of histology when using LCMs. In contrast, PCR provides results that are easy to interpret at one-third the cost of histology [45]. The present study indicated that pooled nPCR was a practical and sensitive technique to detect submicroscopic infections at a population level relative to placental histology. Results also showed that PCR had similar sensitivity and prevalence estimates from either peripheral or placental samples despite the fact that one population analysed included only low-density, submicroscopic infections, suggesting that peripheral blood could provide a viable alternative to placental sampling in challenging environments for prevalence estimation of placental malaria.

Latent class models were used here to provide realistic estimates for sensitivity and specificity of PCR tests for which no true gold standard exists since newer methodologies are substantially more sensitive than the gold standard. PCR is considered to be a more sensitive and specific diagnostic method for malaria in pregnancy than all other available techniques [46]; however, published reports of sensitivity and specificity at a population level are all assessed by comparison to a gold standard [17]. Previous studies in severe clinical cases indicated that sensitivity of PCR for detection of severe *P. falciparum* or *Plasmodium vivax* malaria was 90% or higher, but that specificity may be lower for *P. falciparum* [47] when using a gold standard. Results which were not restricted by assigning a gold standard consistently found PCR to be a more sensitive method than histology across all sets of assumptions evaluated. This analysis allowed additional positive samples detected by PCR to contribute to increased sensitivity of PCR rather than exclusively contribute to specificity calculations. The LCMs produced similar estimates for test characteristics of histology, lower sensitivity and high specificity, as the original analysis of the Mozambican data which had set PCR as the gold standard [13].

The narrow confidence intervals in one-step pools are a direct result of current test characteristics. As the sensitivity and specificity of PCR are quite good, individual testing has a narrower confidence interval than pooled tests (Figure 3A). For tests where characteristics are known to be imperfect, this approach provides an additional benefit in reducing the total number of false positive tests, which narrows confidence intervals [48]. If prevalence of malaria were substantially higher, one-step pooling would require smaller pools and a larger number of tests [49]. However, in low prevalence populations, one-step pooled sampling increases precision of prevalence estimates by increasing pool and sample size given a fixed budget. Optimal pool size depends on individual user objectives: it may be that one would maximize number of pools processed to minimize confidence interval width given fixed resources, or one would minimize number of tests performed given a desired confidence interval width. Findings from the present study based on microscopy-negative samples from pregnant women at delivery were expected to be similar to the general population at low parasite density. Future work will focus on one-step PCR-based pooled sampling in the general population for estimates of malaria prevalence at population level in low transmission areas.

The use of pooled sampling in DNA extraction and PCR greatly reduces workload and cost if test prevalence is low [20, 33, 34]. In the present study, the cost reduction of DNA extraction without reduction of PCR sensitivity was found by pooling five samples in histology-positive individuals and ten samples in histology-negative individuals for the initial screening PCR as in previous work with HIV prevalence estimation [30]. If one-step pools were used in malaria pre-elimination areas, the number of extractions and PCRs performed could be reduced more than ten-fold and still maintain a reasonable precision of population estimates relative to individual sample testing. At a low prevalence, larger pool sizes increase efficiency while maintaining narrow confidence intervals. This would maximize estimate precision with limited surveillance resources. Determination of optimal pool size for each area depends not only on expected prevalence but also on resources available for testing, number of samples available and objective of the testing strategy.

## Conclusions

Pooled nested PCR testing was a sensitive and resource-efficient strategy which would provide sufficient precision for population level estimates of prevalence while requiring limited processing of samples. Latent class models identified test characteristics for PCR, which was substantially more sensitive than histology. Peripheral blood samples could provide an alternative to placental sampling for monitoring prevalence of malaria in pregnancy. Compared to two-step pooling or individual testing, one-step pooling would provide a similar prevalence estimate at the population level. Latent class analysis of test characteristics allows accurate estimation of prevalence using highly sensitive new PCR techniques. PCR-based pooling strategies could be useful for malaria surveys in areas where malaria prevalence is low, submicroscopic infections are common and resources are limited.

## Electronic supplementary material

Additional file 1: **Table of priors used.** Uniform and pessimistic priors used for LCMs of sensitivity and specificity of PCR. Realistic priors for histology used for both models were based on a previous meta-analysis [17]. (PDF 87 KB)

Additional file 2: **Density plots for LCMs using pessimistic PCR priors.** These models use inputs which bias away from high sensitivity and specificity estimates for PCR. Horizontal axis labels represent parameter evaluated, and vertical axis represent density of credible intervals. Dashed black lines represent priors. Overall results using all samples from Malawi and placental samples from Mozambique [13] (thick green lines) compared to result for placental samples only (thin dashed blue lines) or peripheral samples only (thin dashed yellow lines). (PDF 65 KB)

Additional file 3: **Posterior probability table for LCMs using pessimistic priors for PCR characteristics.** For determining assay characteristics, column 1 uses all data from the current Malawi study and placental data from published work in Mozambique [13], column 2 uses only placental data from both countries, and column 3 uses only peripheral data from both countries. Prevalence estimates based on test characteristics are presented in the last two rows. Values presented are median values of posteriors and 95% credible intervals. (PDF 88 KB)

## References

[1] WHO: **Malaria in pregnant women.** http://www.who.int/malaria/areas/high_risk_groups/pregnancy/en/

[2] Steketee RW, Wirima JJ, Hightower AW, Slutsker L, Heymann DL, Breman JG (1996). The effect of malaria and malaria prevention in pregnancy on offspring birthweight, prematurity, and intrauterine growth retardation in rural Malawi. Am J Trop Med Hyg.

[3] Steketee RW, Nahlen BL, Parise ME, Menendez C (2001). The burden of malaria in pregnancy in malaria-endemic areas. Am J Trop Med Hyg.

[4] Slutsker L, Khoromana CO, Hightower AW, Macheso A, Wirima JJ, Breman JG, Heymann DL, Steketee RW (1996). Malaria infection in infancy in rural Malawi. Am J Trop Med Hyg.

[5] Desai M, ter Kuile FO, Nosten F, McGready R, Asamoa K, Brabin B, Newman RD (2007). Epidemiology and burden of malaria in pregnancy. Lancet Infect Dis.

[6] Roll Back Malaria: *The Contribution of Malaria Control to Maternal and Newborn Health*. http://www.rollbackmalaria.org/ProgressImpactSeries/report17.html

[7] Schultz LJ, Steketee RW, Macheso A, Kazembe P, Chitsulo L, Wirima JJ (1994). The efficacy of antimalarial regimens containing sulfadoxine-pyrimethamine and/or chloroquine in preventing peripheral and placental *Plasmodium falciparum* infection among pregnant-women in Malawi. Am J Trop Med Hyg.

[8] WHO (2007). Technical Expert Group Meeting on Intermittent Preventive Treatment in Pregnancy (IPTp).

[9] Menendez C, Bardaji A, Sigauque B, Sanz S, Aponte JJ, Mabunda S, Alonso PL (2010). Malaria prevention with IPTp during pregnancy reduces neonatal mortality. PLoS One.

[10] Eisele TP, Larsen DA, Anglewicz PA, Keating J, Yukich J, Bennett A, Hutchinson P, Steketee RW (2012). Malaria prevention in pregnancy, birthweight, and neonatal mortality: a meta-analysis of 32 national cross-sectional datasets in Africa. Lancet Infect Dis.

[11] Ramharter M, Schuster K, Bouyou-Akotet MK, Adegnika AA, Schmits K, Mombo-Ngoma G, Agnandji ST, Nemeth J, Afene SN, Issifou S, Onnas IN, Kombila M, Kremsner PG (2007). Malaria in pregnancy before and after the implementation of a national IPTp program in Gabon. Am J Trop Med Hyg.

[12] Mockenhaupt FP, Rong B, Till H, Eggelte TA, Beck S, Gyasi-Sarpong C, Thompson WN, Bienzle U (2000). Submicroscopic *Plasmodium falciparum* infections in pregnancy in Ghana. Trop Med Int Health.

[13] Mayor A, Moro L, Aguilar R, Bardaji A, Cistero P, Serra-Casas E, Sigauque B, Alonso PL, Ordi J, Menendez C (2012). How hidden can malaria be in pregnant women? Diagnosis by microscopy, placental histology, polymerase chain reaction and detection of histidine-rich protein 2 in plasma. Clin Infect Dis.

[14] Cohee LM, Kalilani-Phiri L, Boudova S, Joshi S, Mukadam R, Seydel KB, Mawindo P, Thesing P, Kamiza S, Makwakwa K, Muehlenbachs A, Taylor TE, Laufer MK (2014). Submicroscopic malaria infection during pregnancy and the impact of intermittent preventive treatment. Malar J.

[15] Adegnika AA, Verweij JJ, Agnandji ST, Chai SK, Breitling LP, Ramharter M, Frolich M, Issifou S, Kremsner PG, Yazdanbakhsh M (2006). Microscopic and sub-microscopic *Plasmodium falciparum* infection, but not inflammation caused by infection, is associated with low birth weight. Am J Trop Med Hyg.

[16] Okell LC, Bousema T, Griffin JT, Ouedraogo AL, Ghani AC, Drakeley CJ (2012). Factors determining the occurrence of submicroscopic malaria infections and their relevance for control. Nat Commun.

[17] Kattenberg JH, Ochodo EA, Boer KR, Schallig HD, Mens PF, Leeflang MM (2011). Systematic review and meta-analysis: rapid diagnostic tests versus placental histology, microscopy and PCR for malaria in pregnant women. Malar J.

[18] Rogerson SJ, Mkundika P, Kanjala MK (2003). Diagnosis of *Plasmodium falciparum* malaria at delivery: comparison of blood film preparation methods and of blood films with histology. J Clin Microbiol.

[19] Anchang-Kimbi JK, Achidi EA, Nkegoum B, Sverremark-Ekstrom E, Troye-Blomberg M (2009). Diagnostic comparison of malaria infection in peripheral blood, placental blood and placental biopsies in Cameroonian parturient women. Malar J.

[20] Hsiang MS, Lin M, Dokomajilar C, Kemere J, Pilcher CD, Dorsey G, Greenhouse B (2010). PCR-based pooling of dried blood spots for detection of malaria parasites: optimization and application to a cohort of Ugandan children. J Clin Microbiol.

[21] Rantala AM, Taylor SM, Trottman PA, Luntamo M, Mbewe B, Maleta K, Kulmala T, Ashorn P, Meshnick SR (2010). Comparison of real-time PCR and microscopy for malaria parasite detection in Malawian pregnant women. Malar J.

[22] Mockenhaupt FP, Ulmen U, von Gaertner C, Bedu-Addo G, Bienzle U (2002). Diagnosis of placental malaria. J Clin Microbiol.

[23] Ezebialu IU, Eke AC, Ezeagwuna DA, Nwachukwu CE, Ifediata F, Ezebialu CU (2012). Prevalence, pattern, and determinants of placental malaria in a population of southeastern Nigerian parturients. Int J Infect Dis.

[24] Branscum AJ, Gardner IA, Johnson WO (2005). Estimation of diagnostic-test sensitivity and specificity through Bayesian modeling. Prev Vet Med.

[25] Goncalves L, Subtil A, De Oliveira MR, Do Rosario V, Lee PW, Shaio MF (2012). Bayesian latent class models in malaria diagnosis. PLoS One.

[26] Cowling DW, Gardner IA, Johnson WO (1999). Comparison of methods for estimation of individual-level prevalence based on pooled samples. Prev Vet Med.

[27] Sergeant ES, Baldock FC (2002). The estimated prevalence of Johne's disease infected sheep flocks in Australia. Aust Vet J.

[28] Katholi CR, Toe L, Merriweather A, Unnasch TR (1995). Determining the prevalence of *Onchocerca volvulus* infection in vector populations by polymerase chain reaction screening of pools of black flies. J Infect Dis.

[29] White DJ (2001). Vector Surveillance for West Nile Virus. Ann NY Acad Sci.

[30] Tu XM, Litvak E, Pagano M (1994). Studies of AIDS and HIV surveillance. Screening tests: can we get more by doing less?. Stat Med.

[31] Brookmeyer R (1999). Analysis of multistage pooling studies of biological specimens for estimating disease incidence and prevalence. Biometrics.

[32] Chen CL, Swallow WH (1990). Using group testing to estimate a proportion, and to test the binomial model. Biometrics.

[33] Bharti AR, Letendre SL, Patra KP, Vinetz JM, Smith DM (2009). Malaria diagnosis by a polymerase chain reaction-based assay using a pooling strategy. Am J Trop Med Hyg.

[34] Taylor SM, Juliano JJ, Trottman PA, Griffin JB, Landis SH, Kitsa P, Tshefu AK, Meshnick SR (2010). High-throughput pooling and real-time PCR-based strategy for malaria detection. J Clin Microbiol.

[35] Gutman J, Mwandama D, Wiegand RE, Ali D, Mathanga DP, Skarbinski J (2013). Effectiveness of intermittent preventive treatment with sulfadoxine-pyrimethamine during pregnancy on maternal and birth outcomes in Machinga district, Malawi. J Infect Dis.

[36] Ismail MR, Ordi J, Menendez C, Ventura PJ, Aponte JJ, Kahigwa E, Hirt R, Cardesa A, Alonso PL (2000). Placental pathology in malaria: a histological, immunohistochemical, and quantitative study. Hum Pathol.

[37] Menendez C, Ordi J, Ismail MR, Ventura PJ, Aponte JJ, Kahigwa E, Font F, Alonso PL (2000). The impact of placental malaria on gestational age and birth weight. J Infect Dis.

[38] Snounou G, Viriyakosol S, Zhu XP, Jarra W, Pinheiro L, Do Rosario VE, Thaithong S, Brown KN (1993). High sensitivity of detection of human malaria parasites by the use of nested polymerase chain reaction. Mol Biochem Parasitol.

[39] Perandin F, Manca N, Calderaro A, Piccolo G, Galati L, Ricci L, Medici MC, Arcangeletti MC, Snounou G, Dettori G, Chezzi C (2004). Development of a real-time PCR assay for detection of *Plasmodium falciparum*, *Plasmodium vivax*, and *Plasmodium ovale* for routine clinical diagnosis. J Clin Microbiol.

[40] Gelman AR, Donald B (1992). Inference from iterative simulation using multiple sequences. Stat Sci.

[41] Rogan WJ, Gladen B (1978). Estimating prevalence from the results of a screening test. Am J Epidemiol.

[42] Okell LC, Ghani AC, Lyons E, Drakeley CJ (2009). Submicroscopic infection in *Plasmodium falciparum*-endemic populations: a systematic review and meta-analysis. J Infect Dis.

[43] Karl S, Gurarie D, Zimmerman PA, King CH, St Pierre TG, Davis TM (2011). A sub-microscopic gametocyte reservoir can sustain malaria transmission. PLoS One.

[44] Lindblade KA, Steinhardt L, Samuels A, Kachur SP, Slutsker L (2013). The silent threat: asymptomatic parasitemia and malaria transmission. Expert Rev Anti Infect Ther.

[45] Campos IM, Uribe ML, Cuesta C, Franco-Gallego A, Carmona-Fonseca J, Maestre A (2011). Diagnosis of gestational, congenital, and placental malaria in Colombia: comparison of the efficacy of microscopy, nested polymerase chain reaction, and histopathology. Am J Trop Med Hyg.

[46] Poschl B, Waneesorn J, Thekisoe O, Chutipongvivate S, Karanis P (2010). Comparative diagnosis of malaria infections by microscopy, nested PCR, and LAMP in northern Thailand. Am J Trop Med Hyg.

[47] Manning L, Laman M, Rosanas-Urgell A, Turlach B, Aipit S, Bona C, Warrell J, Siba P, Mueller I, Davis TM (2012). Rapid antigen detection tests for malaria diagnosis in severely ill Papua New Guinean children: a comparative study using Bayesian latent class models. PLoS One.

[48] Tu XM, Litvak E, Pagano M (1995). On the informativeness and accuracy of pooled testing in estimating prevalence of a rare disease: application to HIV screening. Biometrika.

[49] Sacks JM, Bolin SR, Crowder SV (1989). Prevalence estimation from pooled samples. Am J Vet Res.

