# Supplementary material for: Pooled PCR testing strategy and prevalence estimation of submicroscopic infections using Bayesian latent class models in pregnant women receiving intermittent preventive treatment at Machinga District Hospital, Malawi, 2010
Source: Malar J. 2014 Dec 18;13:509. doi: 10.1186/1475-2875-13-509 (PMC4301903; doi:10.1186/1475-2875-13-509)
Supplement: Supplementary file 1 — Additional file 1: Table of priors used. Uniform and pessimistic priors used for LCMs of sensitivity and specificity of PCR. Realistic priors for histology used for both models were based on a previous meta-analysis [17]. (PDF 87 KB) [file 12936_2014_3646_MOESM1_ESM.pdf]

**Value**	**Uniform**	**Pessimistic**
Sensitivity Histology (Median, 0.025)	(0.80, 0.50)	(0.80, 0.50)
Sensitivity PCR (Median, 0.025)	(0.5, 0.025)	(0.70, 0.50)
Specificity Histology (Median, 0.025)	(0.90, 0.80)	(0.90, 0.80)
Specificity PCR (Median, 0.025)	(0.5, 0.025)	(0.70, 0.50)
Prevalence Malawi (Median, 0.975)	(0.5, 0.975)	(0.20, 0.45)
Prevalence Mozambique (Median, 0.975)	(0.5, 0.975)	(0.20, 0.45)
