# Supplementary figures and images for: Pooled PCR testing strategy and prevalence estimation of submicroscopic infections using Bayesian latent class models in pregnant women receiving intermittent preventive treatment at Machinga District Hospital, Malawi, 2010
Source: Malar J. 2014 Dec 18;13:509. doi: 10.1186/1475-2875-13-509 (PMC4301903; doi:10.1186/1475-2875-13-509)

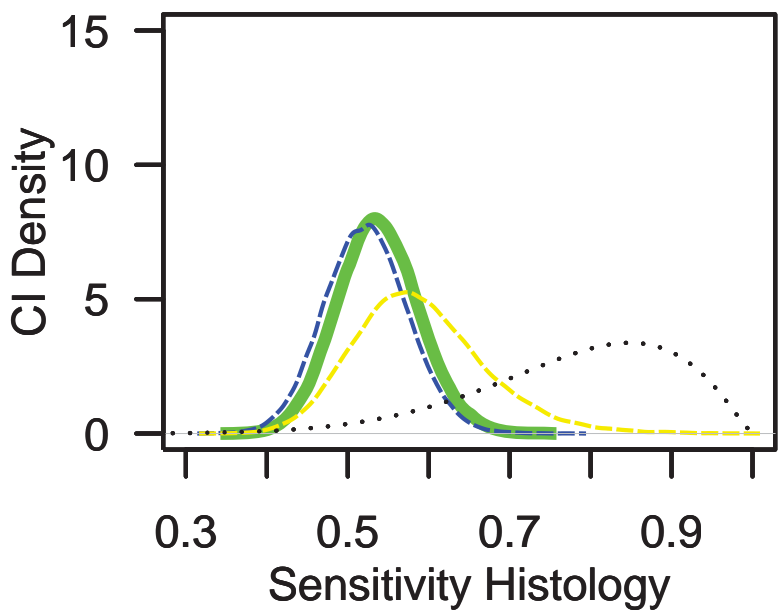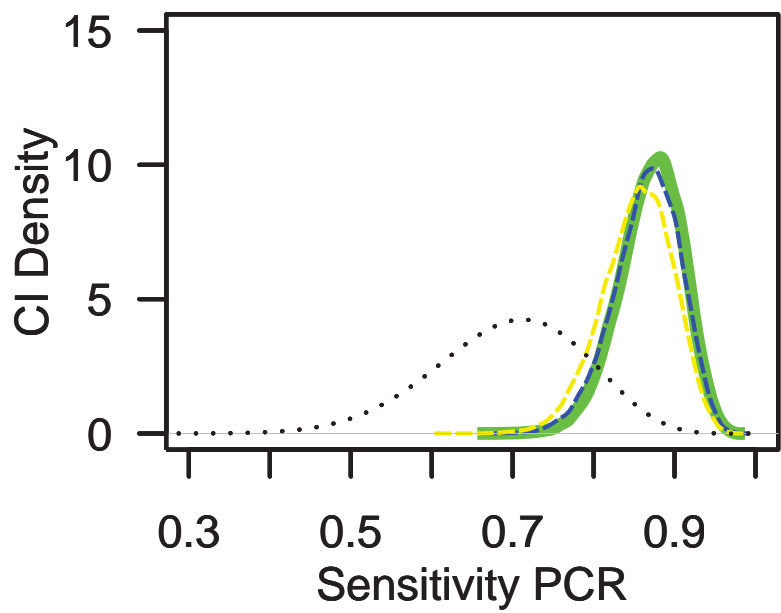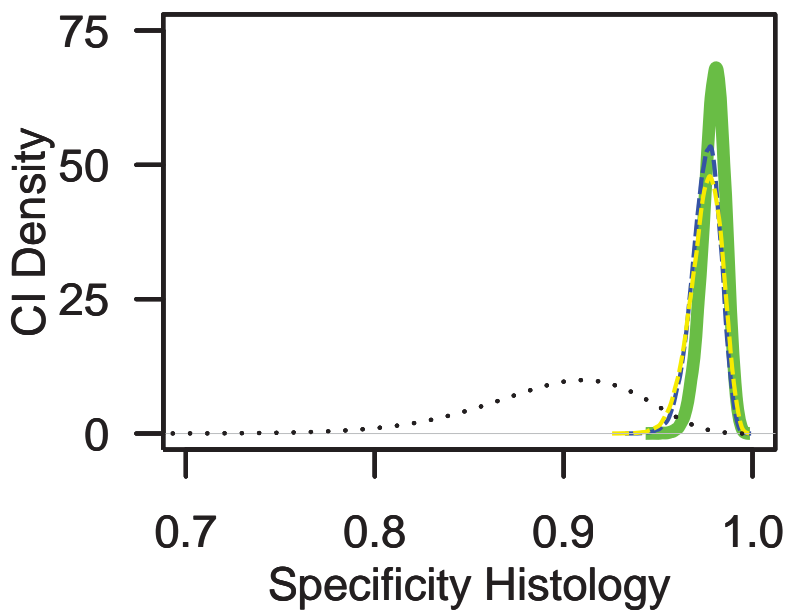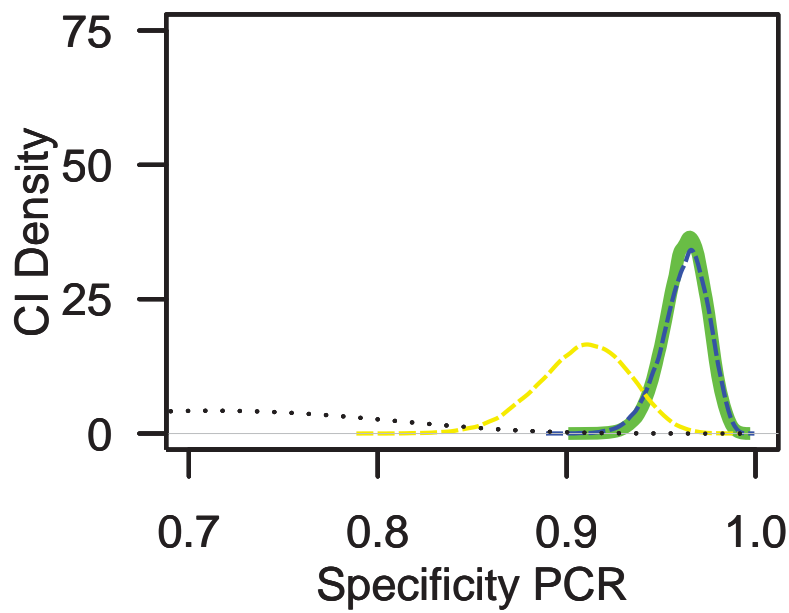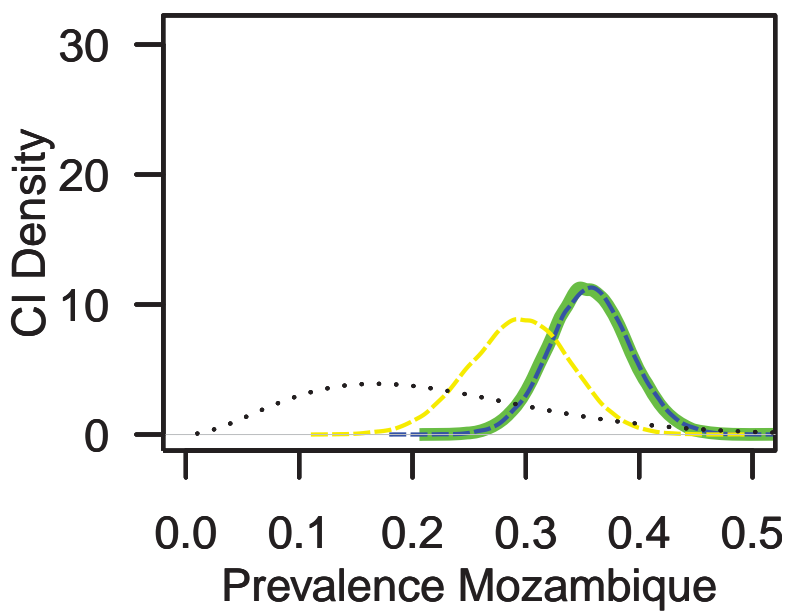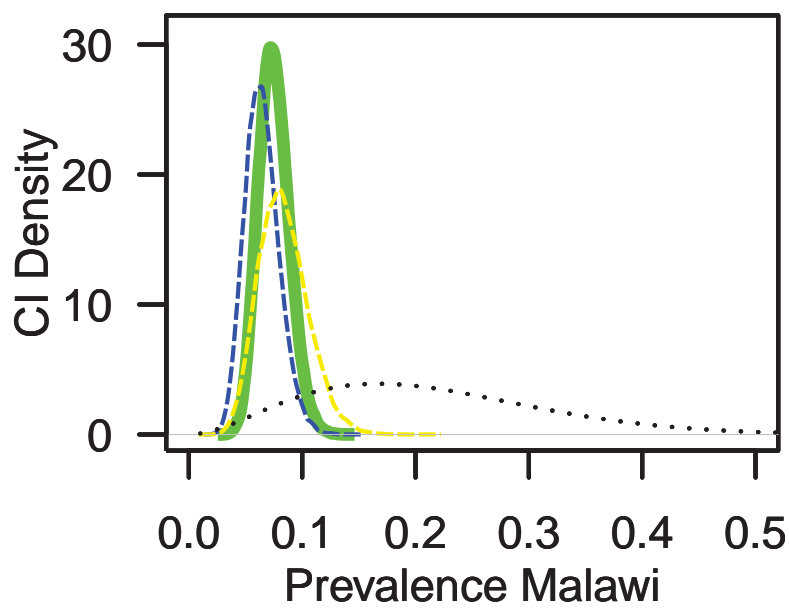

Supplement: Supplementary file 2 — Additional file 2: Density plots for LCMs using pessimistic PCR priors. These models use inputs which bias away from high sensitivity and specificity estimates for PCR. Horizontal axis labels represent parameter evaluated, and vertical axis represent density of credible intervals. Dashed black lines represent priors. Overall results using all samples from Malawi and placental samples from Mozambique [13] (thick green lines) compared to result for placental samples only (thin dashed blue lines) or peripheral samples only (thin dashed yellow lines). (PDF 65 KB) [file 12936_2014_3646_MOESM2_ESM.pdf]
