# Supplementary material for: Pooled PCR testing strategy and prevalence estimation of submicroscopic infections using Bayesian latent class models in pregnant women receiving intermittent preventive treatment at Machinga District Hospital, Malawi, 2010
Source: Malar J. 2014 Dec 18;13:509. doi: 10.1186/1475-2875-13-509 (PMC4301903; doi:10.1186/1475-2875-13-509)
Supplement: Supplementary file 3 — Additional file 3: Posterior probability table for LCMs using pessimistic priors for PCR characteristics. For determining assay characteristics, column 1 uses all data from the current Malawi study and placental data from published work in Mozambique [13], column 2 uses only placental data from both countries, and column 3 uses only peripheral data from both countries. Prevalence estimates based on test characteristics are presented in the last two rows. Values presented are median values of posteriors and 95% credible intervals. (PDF 88 KB) [file 12936_2014_3646_MOESM3_ESM.pdf]

Value	Pessimistic Overall	Pessimistic Placental	Pessimistic Peripheral
Sensitivity Histology (Median, CI)	0.537 (0.442, 0.639)	0.522 (0.425, 0.626)	0.582 (0.450, 0.769)
Sensitivity nPCR (Median, CI)	0.873 (0.787, 0.938)	0.868 (0.777, 0.936)	0.857 (0.762, 0.930)
Specificity Histology (Median, CI)	0.980 (0.967, 0.990)	0.976 (0.959, 0.988)	0.976 (0.956, 0.990)
Specificity nPCR (Median, CI)	0.963 (0.939, 0.982)	0.964 (0.935, 0.983)	0.910 (0.859, 0.952)
Prevalence Mozambique (Median, CI)	0.35491 (0.287 0.425)	0.357 (0.288, 0.428)	0.295 (0.205, 0.380)
Prevalence Malawi (Median, CI)	0.07432 (0.051, 0.103)	0.064 (0.038, 0.097)	0.082 (0.046, 0.130)
